# Neural correlates of inhibitory control and associations with cognitive outcomes in Bangladeshi children exposed to early adversities

**DOI:** 10.1111/desc.13245

**Published:** 2022-02-22

**Authors:** Eileen F. Sullivan, Wanze Xie, Stefania Conte, John E. Richards, Talat Shama, Rashidul Haque, William A. Petri, Charles A. Nelson

**Affiliations:** ^1^ Labs of Cognitive Neuroscience, Division of Developmental Medicine Boston Children's Hospital Boston USA; ^2^ Harvard Graduate School of Education Cambridge USA; ^3^ School of Psychological and Cognitive Sciences Peking University Beijing China; ^4^ PKU‐IDG/McGovern Institute for Brain Research Peking University Beijing China; ^5^ Department of Psychology University of South Carolina Columbia USA; ^6^ ICDDR, B Dhaka Bangladesh; ^7^ Infectious Diseases & International Health University of Virginia Charlottesville USA; ^8^ Harvard Medical School Boston USA

**Keywords:** cognitive development, early adversity, event‐related potentials, inhibitory control, poverty, source analysis

## Abstract

There is strong support for the view that children growing up in low‐income homes typically evince poorer performance on tests of inhibitory control compared to those growing up in higher income homes. Unfortunately, the vast majority of the work documenting this association has been conducted in high‐income countries. It is not yet known whether the mechanisms found to mediate this association would generalize to children in low‐ and middle‐income countries, where the risks of exposure to extreme poverty and a wide range of both biological and psychosocial hazards may be greater. We examined relations among early adversity, neural correlates of inhibitory control, and cognitive outcomes in 154 5‐year‐old children living in Dhaka, Bangladesh, an area with a high prevalence of poverty. Participants completed a go/no‐go task assessing inhibitory control and their behavioral and event‐related potential responses were assessed. Cortical source analysis was performed. We collected measures of poverty, malnutrition, maternal mental health, psychosocial adversity, and cognitive skills. Supporting studies in high‐income countries, children in this sample exhibited a longer N2 latency and higher P3 amplitude to the no‐go versus go condition. Unexpectedly, children had a more pronounced N2 amplitude during go trials than no‐go trials. The N2 latency was related to their behavioral accuracy on the go/no‐go task. The P3 mean amplitude, behavioral accuracy, and reaction time during the task were all associated with intelligence‐quotient (IQ) scores. Children who experienced higher levels of psychosocial adversity had lower accuracy on the task and lower IQ scores.

## 1 INTRODUCTION

Executive functions (EF) encompass high‐level cognitive processes that serve as essential building blocks for children's cognitive development, academic achievement, and well‐being (Ahmed et al., 2019; Blair & Razza, 2007; Morgan et al., 2019). Inhibitory control, a core EF skill related to the ability to resist impulsive tendencies and behaviors, is predictive of children's school readiness and social‐emotional skills (Carlson & Wang, 2007; Mann et al., 2017; Rhoades et al., 2009). Associations between socioeconomic status (SES) and inhibitory control have been reliably documented in children, suggesting that inhibitory control is sensitive to environmental inputs (Raver et al., 2013; St. John et al., 2019; Vrantsidis et al., 2020). It is essential to develop a mechanistic understanding of how environmental factors related to SES impact inhibitory control, given the importance of inhibitory control abilities to children's success and well‐being. Discovery of these mechanisms could facilitate the early identification of risk factors and deficits in cognitive functions, informing the design of effective interventions.

Numerous studies have sought to understand what proximal aspects of children's environments may be driving the association between social class and inhibitory control. Several characteristics of the early environment have been found to partially explain the link between SES and inhibition, including parental responsivity, predictability, language exposure, and cognitive stimulation in the home (Hackman et al., 2015; Lecheile et al., 2020; Sarsour et al., 2011). Findings from neuroimaging studies have helped to uncover neural mechanisms of inhibitory control and deepen our understanding of how early experiences shape the development of inhibition (Abdul Brydges et al., 2014; Jonkman, 2006; Rahman et al., 2017). For instance, Swingler et al. (2018) found that maternal emotional support was related to preschoolers’ brain activity during an inhibitory control task, as indexed by the N2 event‐related potential (ERP) component.

The existing evidence base on drivers of SES‐based differences in inhibition is both informative and compelling. However, the vast majority of studies providing this evidence were conducted in high‐income countries (HICs) (Haslam et al., 2019; Lund et al., 2020). It is not yet known whether the mechanisms identified in studies of SES in HICs are generalizable to children growing up in extreme poverty in low‐ and middle‐income countries (LMICs). Children in LMICs are more likely than their HIC‐based peers to be chronically exposed to a broad range of adverse biological factors (e.g., malnutrition, toxin exposure) and psychosocial experiences (e.g., abuse, neglect) that can affect brain structure and function (Benjet, 2010; Donowitz et al., 2018; Lu et al., 2020; Nelson & Gabard‐Durnam, 2020). More evidence from LMICs is needed to explore whether adverse factors associated with extreme poverty uniquely impact the neural and behavioral basis of inhibitory control.

**Research Highlights**

- Children living in extreme poverty are exposed to hazards that could compromise inhibitory control skills, yet the underlying neural mechanisms of these impacts are underexplored.
- We measured Bangladeshi children's event‐related potentials and behavioral performance during a go/no‐go task to understand the relations between early adversity and correlates of inhibition.
- Children exhibited lower accuracy and a more pronounced P3 amplitude for trials requiring behavioral inhibition (no‐go condition) than trials requiring no inhibition (go condition).
- The N2 latency was related to higher accuracy on the go/no‐go task. Higher levels of psychosocial adversity were associated with lower accuracy and IQ scores.

Children living in extreme poverty often do not experience just one form of adversity in isolation (Black et al., 2017). A major challenge to investigating associations between adversity on inhibitory control in LMICs is disentangling the unique consequences of co‐occurring risk factors (e.g., malnutrition, abuse, safety risks). Behavioral measures of EF may not be sensitive enough to capture the distinct impacts of multiple adverse factors on inhibitory control. Electrophysiological measures such as electroencephalography (EEG) and ERPs are uniquely positioned to detect how adverse factors are related to the neural mechanisms underlying inhibitory control. There is a need to utilize objective, scalable and (relatively) culture‐free tools in LMICs to investigate relations between early adverse experiences and inhibition. The recordings of EEG activity and the measure of ERPs meet these needs.

Exposure to biological and psychosocial adversity has been reliably linked to consequences on overall brain and cognitive development in LMICs (Jensen et al., 2019; Jensen et al., 2021; Tarullo et al., 2017; Wijeakumar et al., 2019). To develop concrete strategies of prevention and intervention, the mechanisms underlying associations between adversity and development must be understood. Specifically, it is key to identify which aspects of adversity could be impacting the brain structures and functions that subserve the development of inhibitory control. Studies have suggested that disruption to children's expectable psychosocial environments can be linked to the neural and behavioral correlates of inhibition. Psychosocial adversity, encompassing abuse, neglect, family mental illness, and other significant stressors, has been shown to be related to inhibitory control (Cowell et al., 2015; Gueron‐Sela et al., 2018; McDermott et al., 2012). For instance, children experiencing profound early psychosocial deprivation were found to exhibit slower neural responses and diminished accuracy during an ERP go/no‐go task (assessing inhibitory control) as compared to children who had been placed in foster care or had never been institutionalized (McDermott et al., 2012). A follow‐up study of this same sample found that children who remained in neglectful environments performed more poorly than those removed from neglectful situations and placed into family environments on behavioral EF outcomes at 16 years of age, indicating the potentially long‐lasting impacts of extreme neglect (Wade et al., 2019). These findings demonstrate the profound effects of the caregiving environment on the development of inhibition and also highlight the efficacy of employing neuroimaging methods to elucidate mechanisms of the effects of early adversity.

Exposure to maltreatment (e.g., abuse, violence) is another aspect of psychosocial experience that is negatively associated with inhibitory control skills, although further investigation is needed to explore the mechanisms and generalizability of this association (Lund et al., 2020). Associations between child maltreatment and inhibitory control were shown in 3‐ to 9‐year‐old children in the United States; children who experienced maltreatment in infancy or chronically throughout childhood showed poorer performance on inhibitory control tasks (Cowell et al., 2015). In another study of 12‐ to 13‐year‐olds in the US, children who had experienced maltreatment showed atypical ERP responses and lower accuracy during an inhibitory control task as compared to nonmaltreated peers (Bruce & Kim, 2020). Additional studies are needed to investigate whether similar associations between maltreatment and inhibitory control are evident in LMIC populations.

Maternal mental health could also shape children's psychosocial experiences and affect their inhibitory control skills. In one longitudinal study of children in the United States, maternal depression was predictive of preschoolers’ performance on inhibitory control and working memory tasks (Gueron‐Sela et al., 2018). One study in China found that children whose mothers had higher stress levels tended to perform more poorly on inhibitory control tasks at 2 years of age (Liu et al., 2018). These findings, along with others (Hughes et al., 2013; Lund et al., 2020), suggest that maternal mental health is a key feature of the early psychosocial environment that could influence children's inhibitory control abilities. Yet, the neural mechanisms underlying this association are still largely unexplored, especially in LMICs. Importantly, different forms of psychosocial adversity, including neglect, maltreatment, and maternal mental health issues, could co‐occur, but further study of the possible interactive effects of these experiences on inhibition and cognition more broadly is needed.

Along with these potential pathways linking children's psychosocial experiences to inhibitory control, biological risks could also potentially impact children's development of inhibition. Malnutrition is extremely common in many LMICs, and is known to have consequences on children's cognitive development (Ajayi et al., 2017; McCoy et al., 2015). Evidence from interventions suggests a causal link between nutrition and cognitive outcomes, as nutrition supplementation in various forms has been shown to lead to improvements in children's cognitive abilities (Black et al., 2015; Larson & Yousafzai, 2017). Supplementing the behavioral evidence on the pathways linking malnutrition and EF, neuroimaging studies have uncovered possible mechanisms by which malnutrition could impact brain and cognitive development. Micro‐ and macronutrient deficiencies can lead to basic insults on early brain development, leading to persistent effects on brain architecture and functioning (Prado & Dewey, 2014; Turesky et al., 2020). One study in the United States found that nutrition supplementation during the first year of life was associated with differences in ERP responses during an inhibitory control task at 5 years of age (Liao et al., 2017). The consequences of extreme malnutrition on the neural processes underlying inhibitory control have yet to be examined explicitly in LMICs. Furthermore, the impact of co‐occurring biological and psychosocial adversities on inhibition should be explored to reflect the reality that many children experience a complex set of risk factors and protective factors simultaneously.

The current evidence base on the impact of biological and psychosocial exposures serves as an important foundation for understanding children's cognitive development. However, there is a need for further investigation of how early adversity may affect the development of inhibitory control in LMICs. Children living in extreme poverty are at risk of experiencing many forms of adversity simultaneously (Black et al., 2017; Hurley et al., 2016). Therefore, mechanistic evidence on the impact of multiple, potentially interacting adversities is needed to understand which factors have the most profound effects on brain development.

The current study sought to address these gaps in the field by examining how co‐occurring risk factors are related to neural and behavioral correlates of inhibitory control in children living in an impoverished area of Dhaka, Bangladesh. The participants completed study visits at 3 years old and were followed longitudinally at 5 years old. We collected data on SES, maternal mental health, nutrition status, and psychosocial adversity. A cognitive assessment was also conducted. Inhibitory control was assessed when participants were 5 years old using a go/no‐go ERP task, which required children to inhibit behavioral responses to a target stimulus, while EEG data was recorded. The relations among biological and psychosocial risks, ERP components implicated in inhibitory control, behavioral performance on the go/no‐go task, and cognitive abilities were investigated.

Go/no‐go ERP tasks are commonly used in developmental studies (Loman et al., 2013; McDermott et al., 2012, St. John et al., 2019) and offer multiple neural markers to study in relation to early experience and cognitive outcomes. ERPs are electrophysiological signals that are generated in response to a stimulus or task and can index specific aspects of cognitive processing. In this study, we focus on two widely studied ERP components: the N2 and P3. In both children and adults, the N2 peak amplitude and latency (time between stimulus onset and N2 peak amplitude) have been implicated in focused attention, inhibition, and cognitive control; the P3 mean amplitude has been implicated in information processing and inhibitory control (Brydges et al., 2014; Downes et al., 2017; Kopp et al., 2020). Given their role in inhibition and the relations found between early adversity and inhibition, the N2 and P3 could plausibly as objective and sensitive indices of the impact of early adversity on neural circuitry underlying inhibitory control. However, the extent to which the N2 and P3 responses are generalizable across contexts must first be explored. Typically, the N2 has been shown to be generated by the anterior cingulate cortex (ACC) and prefrontal regions (Bekker et al., 2005; Bokura et al., 2001). The P3 has been localized to precentral and parietal regions (Bokura et al., 2001; Huster et al., 2009). We seek to identify whether the same neural generators of the N2 and P3 are evident in children growing up in an LMIC and infer which brain regions may be driving the association between adversity and inhibitory control skills.

We sought to address three main aims in this study. First, we aimed to assess whether neural markers previously found to underlie inhibitory control are generalizable to our study context by examining the N2 and P3 components. Specifically, we conducted source analysis to probe whether these components are generated from the expected brain regions. Second, to explore potential brain‐behavior relationships in this setting, we investigated whether ERP responses are related to behavioral performance on the go/no‐go task. We also explored whether ERP and behavioral responses during the go/no‐go task are related to overall cognitive functioning, indexed by intelligence quotient (IQ) scores. Finally, to study how early adversity may affect inhibitory control abilities, we investigated whether early adverse factors are predictive of children's ERP responses, behavioral performance on the go/no‐go task, or IQ. We hypothesized that children who experience more extreme adversity would show less typical ERP responses, exhibit poorer inhibitory control, and have lower IQ scores. With the current study, we build off and extend prior evidence on the development of inhibitory control by investigating whether established neural mechanisms of inhibition operate similarly in children growing up in a setting of extreme poverty.

## 2 METHODS

### 2.1 Participants

Participants live in Dhaka, Bangladesh, and were drawn from two longitudinal cohorts of the Bangladesh Early Adversity Neuroimaging (BEAN) project. One cohort of 130 children (58 females) were originally part of an oral vaccine efficacy trial and were later recruited for the BEAN study at 36 months of age. A second cohort of 80 children (31 females) with a slightly higher average income level were recruited specifically for the BEAN study at 36 months of age. The two cohorts had overlapping distributions of SES and the children were living in the same neighborhood, so all children were treated as one continuous cohort with regard to their SES. All participants had several measures of early adversity collected at 36 and 60 months old and underwent ERP and cognitive tests at 60 months old (*M *= 62.42, *SD *= 2.25). Inclusion criteria limited the sample to children born > = 34 weeks gestational age with no history of neurological abnormalities or traumatic brain injury, genetic disorders, or visual or auditory impairments. The final sample for the ERP analyses included 154 children who provided usable ERP data after artifact detection and rejection. Ethical approval was obtained from research and ethics review committees at The International Centre for Diarrhoeal Disease Research, Bangladesh and the Institutional Review Board at Boston Children's Hospital. Data were collected in a medical clinic in the Mirpur neighborhood of Dhaka, where local staff were fully trained in behavioral and EEG data collection.

### 2.2 Stimuli and task procedure

**FIGURE 1:**
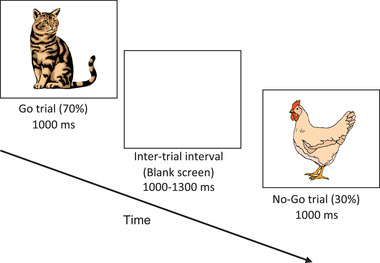
The go/no‐go paradigm. *Note*. Each trial of the paradigm consisted of a picture of an animal displayed on a white background for 1000 ms, followed by a blank screen for 1000–1300 ms. 70% of trials were go trials (cat, dog, cow) and 30% of trials were no‐go trials (chicken)

EEG was collected at the 60‐month study visit while children sat approximately 65 cm from a monitor in a dimly lit room and viewed a go/no‐go paradigm. Stimuli and task procedure are shown in Figure 1. Images were presented using E‐Prime 2.0 (Psychological Software products, Harrisburg, PA, USA). The stimuli used in the go/no‐go task consisted of images of four animals common in Bangladesh presented on a white background. Children were told to press a button when they saw a cat, dog, or cow (go trials), but not when they saw a chicken (no‐go trials). Children first completed 15 practice trials with feedback and 15–30 trials with no feedback. Next, the task consisted of 170 trials with no feedback, of which 70% were go trials and 30% were no‐go trials. Each trial consisted of the image of an animal presented for 1000 ms, followed by a blank white screen presented for 1000–1300 ms (randomized). Participants’ looking behavior was monitored and recorded using a Tobii X2‐60 eye‐tracking system. Behavioral responses (button presses) during the paradigm were recorded. Average accuracy was calculated for all trials and each condition separately. Mean reaction time was computed for correct go trials and incorrect no‐go trials, as these are the trials for which the child pressed the button.

### 2.3 EEG data collection, processing, and analysis

Continuous EEG was recorded while participants viewed the paradigm using a 128‐channel HydroCel Geodesic Sensor Net (HGSN) with a NetAmps 300 high‐input amplifier (Electrical Geodesic Inc., Eugene, OR). Impedances were limited to a maximum of 100 kΩ and data was sampled at 500 Hz from all electrodes. EEG data was filtered with a 0.3–30 Hz finite impulse response (FIR) bandpass filter in ERPLAB (Lopez‐Calderon & Luck, 2014). The recordings were segmented into epochs from 100 ms before stimulus onset to 900 ms following stimulus onset. Segmented data was visually inspected and epochs containing artifacts due to eye‐movements, eye‐blinks, or drift were rejected. Segmented data was also automatically removed in ERPLAB using absolute or stepwise criteria (EEG amplitude < −100 or > 100 μV or change in EEG > 100 μV in a 100 ms moving window with 50 ms steps). Individual electrodes with a voltage change exceeding 200 μV were rejected. Epochs were automatically rejected if 18 or more electrodes were removed due to voltage change. For trials with fewer than 18 electrodes removed, individual electrodes were linearly interpolated with the five closest usable electrodes weighted by their distances to the bad electrode (Xie & Richards, 2016; Xie & Richards, 2017). Data were referenced to an average reference after artifact rejection and channel interpolation. Only go and no‐go trials with correct behavioral responses were used in analyses of the stimulus‐locked ERP components. The average number of acceptable trials was 89.29 (*SD* = 13.68) for the go condition and 31.81 (*SD* = 7.59) for the no‐go condition. An equal number of acceptable trials for each condition were randomly selected (e.g., including a random subset of acceptable go trials to match the number of acceptable no‐go trials). Participants’ data was removed from further analysis if there were fewer than 10 acceptable trials for each condition. Out of the 210 participants originally enrolled, 154 participants contributed acceptable ERP data and were therefore included in final analyses. Of the 56 participants who were not included, 34 did not participate in EEG, 14 had technical issues during data collection, and eight did not have enough included trials per condition.

Data from multiple electrodes were averaged into seven clusters to assess ERPs from regions commonly associated with EF (Table S1). The mean P3 amplitude was defined as the average response from 400 to 700 ms for each condition and was calculated for the Parietal_Left, Parietal_Z, Parietal_Right, and Central_Z clusters. Peak N2 amplitude, defined as the peak response from 250 to 500 ms for each condition, and latency to peak N2 amplitude were calculated for the Frontal_Left, Frontal_Z, Frontal_Right, and the Central_Z clusters. We used the mean amplitude for the P3 component because many children at this age do not have a clear P3 peak. In contrast, the peak of the N2 component at this age is sharp and easy to identify. Thus, peak amplitudes provide a comparison between experimental conditions at their maxima. A similar approach has been implemented in previous ERP studies investigating perceptual and cognitive processes in young children, that is, using the peak amplitude and latency for the N290 component but mean amplitude for the P400 and Nc components (Xie et al., 2019). The normalized difference between conditions, calculated for each ERP component as the difference between the no‐go and go condition divided by the mean of the two conditions, was used when testing associations between ERP components and adversity measures or behavioral outcomes.

### 2.4 Cortical source analysis

**FIGURE 2:**
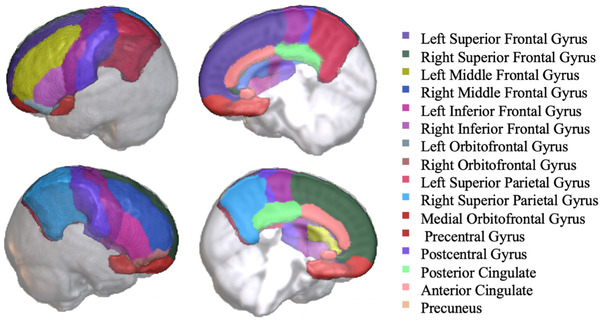
Atlas of 16 regions of interest displayed on the age‐appropriate MRI template

Cortical source analysis was computed using Fieldtrip (Oostenveld et al., 2011) functions to solve the forward and inverse problems. Custom MATLAB scripts (adapted from https://osf.io/knf9t/, Conte & Richards, 2021a) were implemented to obtain the distribute current source density and perform data reduction to a set of selected regions of interest (ROIs) implicated in inhibitory control tasks (Bekker et al., 2005; Bokura et al., 2001). Figure 2 displays the set of ROIs selected for the current study. Realistic head models were created using individual MRIs when available (*n *= 78), whereas MRIs close in size to the participants’ head were utilized when own imaging data was unavailable (*n *= 76). MRIs were acquired at the National Institute for Neuroscience and Hospital (NINSH) in Dhaka, Bangladesh using a 3 T Siemens MAGNETOM Verio scanner. Structural T1‐weighted magnetization‐prepared rapid gradient‐echo (MPRAGE) scans were acquired with the following parameters: TR = 2500 ms, TE = 3.47 ms, 176 sagittal slices, 1 mm^3^ voxels, FOV = 256 mm.

All MRIs were segmented into nine tissue types (i.e., gray matter, white matter, cerebrospinal fluid, dura, skull, muscle, eyes, nasal cavity, and scalp) to provide an accurate description of the head's compartments (Conte et al., 2020; Gao et al., 2019; Guy et al., 2016; Xie & Richards, 2017). We generated tetrahedrons wireframe grids and a finite element method (FEM) head model with source dipoles restricted to the gray matter and eyes (Conte & Richards, 2021b). Electrode placements were defined based on the coordinates of five fiducial markers placed on the scalp using the pictures of each participant's net placement. Then, the coordinates of all electrodes were reconstructed and coregistered with the MRI. The inverse solution was estimated using the exact‐LORETA method (eLORETA; Pascual‐Marqui, 2007; Pascual‐Marqui et al., 2011) and applied to the ERP dataset. A singular value decomposition (SVD) was performed at each location of the source model to obtain a measure of the reconstructed source at each timepoint of the ERP segment. SVD values of both experimental conditions were then averaged within each ROI. For visualization purposes, the individual source solutions were normalized to an age‐appropriate MRI template. The source activity at the peak of each component was averaged across participants and displayed on the MRI template space.

### 2.5 Measures of early adversity

#### 2.5.1 Poverty

Household poverty was assessed from items on a sociodemographic questionnaire administered at 36 months of age. A cumulative sum score was constructed to measure household poverty. This score included (1) income‐to‐needs ratio; (2) housing risks and assets as observed during a home visit including flooring materials, wall materials, roof materials, presence of cooking gas, toilet type, private versus shared toilet, open drain in from of house, municipality provided water supply, and crowding (> 3 people per room); and (3) a count of common family assets noted by an observer during a home visit including presence of a bed, table, chair, bench, phone, clock, radio, television, bicycle, motorcycle, sewing machine, and fan. The average daily income was $2.18 (*SD* = 2.11) per household member, with 64% of families living in extreme poverty, defined by the World Bank as living on less than $1.90 per person per day. See Table S2 for full descriptive statistics.

#### 2.5.2 Malnutrition

To assess malnutrition, children's height was measured at 36 months of age and used to calculate their height‐for‐age z‐score (HAZ) using the World Health Organization's Anthro Plus software (version 3.2.2). A subset of participants (*n* = 104) had HAZ measured at 60 months of age as well; HAZ was highly correlated between the 36‐month and 60‐month timepoint (*r*(104) = 0.90, *p* < 0.001). Children had an average HAZ of −1.33 (*SD* = 1.04) at 36 months of age, and 26% of children met the criteria for stunting (HAZ < −2).

#### 2.5.3 Maternal mental health

Mothers’ level of psychological stress and depression was measured when participants were 36 months old using a cumulative summary score of three questionnaires: the Edinburgh Postnatal Depression Scale (EPDS) (Gausia et al., 2007), the Perceived Stress Scale (PSS) (Cohen et al., 1983), and the Tension Scale (Karasz et al., 2013). The Bengali version of the EPDS was validated in a Bangladeshi sample (Gausia et al., 2007) and was used in this study after the local psychologists added select colloquial terminologies as options for administration. The PSS was translated to Bengali by two bilingual psychologists separately. The translations were reviewed by a team of experts and nonstudy mothers; a first version of the translated PSS was field tested, modified to clarify points of confusion reported by mothers, and back translated and matched to the original version. Pretesting of the Bengali version of the PSS was conducted with 14 mothers and showed a test‐retest reliability score after a 7‐day gap was *r* = 0.86. The Tension Scale was developed specifically for Bangladeshi women (Karasz et al., 2013) and was used in the current study with no further modifications. The three questionnaires were highly interrelated in this sample (Cronbach's α = 0.721), and therefore scores on the three scales were summed to create a summary score encapsulating overall mental health and well‐being. The average maternal stress summary score was 52.26 (*SD* = 17.23).

#### 2.5.4 Psychosocial adversity

Children's cumulative exposure to adverse psychosocial experiences was assessed using the Childhood Psychosocial Adversity Scale (CPAS), a parent‐reported questionnaire developed specifically for the study population, when children were 60 months old (Berens et al., 2019). The CPAS was informed by qualitative work with Bangladeshi mothers and field workers, designed in collaboration with local psychologists, and pretested in the current study setting. The questionnaire includes nine subscales: harsh discipline and abuse, neglect, caregiver emotional availability, depression, social isolation, physical intimate partner violence, verbal abuse and family conflict, household economic stress, and community adversity. The CPAS has been shown to have good internal consistency for all subscales in the BEAN sample (Cronbach's alpha > 0.7). For the purpose of these analyses, the full‐scale score (simple sum of all subscales) was used as an index of cumulative exposure. The average score on the CPAS was 39.33 (*SD* = 21.66).

### 2.6 Cognitive assessment

Children's cognitive development was assessed by local psychologists when children were 60 months of age using the Wechsler Preschool and Primary Scale of Intelligence, Third Edition (WPPSI‐III) (Wechsler, 1991). The WPPSI was adapted to the Bangladeshi context and has been shown to be locally acceptable (Aboud, 2007). For the current analyses, full‐scale IQ was used as a measure of overall higher‐order cognitive functioning. Full‐scale IQ was strongly correlated with both performance IQ (*r*(152) = 0.88, *p* < 0.001) and verbal IQ subscales (*r*(152) = 0.85, *p* < 0.001). Children's mean full‐scale IQ score on the WPPSI was 86.94 (*SD* = 9.33).

### 2.7 Statistical approach

To address study aim one, ERP components of interest (N2 peak amplitude and latency, P3 mean amplitude) were analyzed using a repeated‐measures analyses of variance (RM‐ANOVAs) were performed with condition and electrode cluster as within‐subject factors. Greenhouse‐Geisser corrections were applied when necessary to control for violations of sphericity. Post‐hoc t‐testing was performed to investigate which electrode clusters revealed significant condition differences. Bonferroni corrections for multiple comparisons were performed. *T* tests were used to assess condition‐differences (go vs. no‐go) in reaction time and accuracy.

To address study aim two, Pearson correlations were used to investigate possible associations among ERP components, reaction time, accuracy, and IQ. Multiple linear regressions were used to assess study aim three concerning whether measures of early adversity were related to ERP components of interest, behavioral performance on the go/no‐go task, or IQ. RM‐ANOVAs were performed using IBM SPSS Statistics (version 27, IBM Corp, Armonk, NY, USA). Correlations and regressions were performed using RStudio (version 1.3.1093, Boston, MA, USA).

## 3 RESULTS

### 3.1 Behavioral performance

**FIGURE 3:**
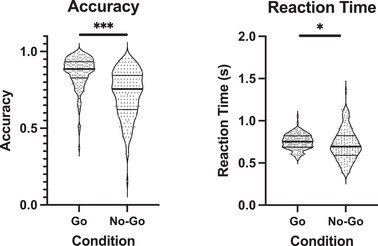
Violin plots of behavioral performance on the go/no‐go task. *Note*. Thick black lines represent mean accuracy or reaction time for each condition. Thin black lines represent the 25^th^ and 75^th^ percentile. Gray triangles represent individual participants’ data points. * *p* < 0.05; ***p < 0.001

In line with prior studies, children had higher mean accuracy for go trials (86.40%) than no‐go trials (72.57%), *t*(277.48) = 9.49, p < 0.001. Children's average reaction time was faster for incorrect trials (0.71 s) than correct trials (0.75 s), *t*(220.51) = 2.34, *p* = 0.02 (see Figure 3).

### 3.2 ERP responses

#### 3.2.1 N2 findings

**FIGURE 4:**
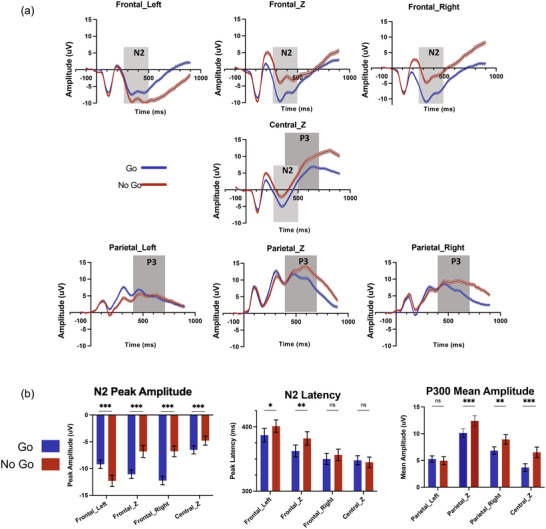
ERP grand‐averaged waveforms and responses to the go and no‐go conditions. *Note*. Panel a: Grand‐averaged ERP waveform for the go (blue) and no‐go (red) condition for midline electrode clusters. The N2 was analyzed in frontal and Central_Z clusters. The P3 was analyzed in parietal and Central_Z clusters. Gray boxes represent time windows for each component. Solid lines represent the grand average of all participants. Dotted lines and shading represent +/‐ 1 standard error. Panel b: Average ERP responses to go (blue) and no‐go (red) conditions for each electrode cluster tested. Bars represent 95% confidence intervals. * *p* < 0.05; **p < 0.01; ***p < 0.001, all Bonferroni‐corrected

The RM‐ANOVA of the N2 peak amplitude revealed significant main effects of electrode cluster, *F*(2.48, 379.05) = 48.81, *p* < 0.001, and go versus no‐go condition, *F*(1, 153) = 453.17, *p* < 0.001 . A significant interaction effect between electrode cluster and condition was also found, *F*(2.51, 384.25) = 110.86, *p* < 0.001 (see Figure 4). After correcting for multiple comparisons, the N2 peak amplitude in Frontal_Z (midline), Frontal_Right, and Central_Z electrode clusters was significantly greater (more negative) for the go condition than the no‐go condition (*p*‐values < 0.001). In contrast, the N2 peak amplitude in the Frontal_Left cluster was significantly more negative for the no‐go condition compared to the go condition (*p* < 0.001). Because all clusters showed significant condition differences, the difference between the go and no‐go N2 peak amplitude in these four clusters were averaged together and used in further analyses.

For the N2 peak latency, an RM‐ANOVA revealed a significant main effect of electrode cluster, *F*(2.68, 410.71) = 44.70, *p* < 0.001, significant main effect of condition, *F*(1, 153) = 8.06, *p* = 0.005, and significant condition X electrode cluster interaction effect *F*(3, 459) = 4.64, *p* = 0.003 (see Figure 4). Post‐hoc tests revealed that, after correction for multiple comparisons, there was a significant condition difference in the Frontal_Left and Frontal_Z electrode clusters (*p* = 0.039 and 0.004, respectively), with longer latencies for the no‐go trials, but no significant condition difference in either the Frontal_Right or Central_Z clusters. Therefore, the average of the Frontal_Left and Frontal_Z N2 latency (difference between go and no‐go latencies) was used in subsequent analyses.

#### 3.2.2 P3 findings

For the P3 mean amplitude, the RM‐ANOVA results showed a significant main effect of electrode cluster (*F*(2.69, 411.80) = 98.74, *p* < 0.001) and condition (*F*(1, 153) = 42.62, *p* < 0.001), in addition to a significant electrode cluster X condition interaction, *F*(2.86, 437.07) = 18.95, *p* < 0.001 (see Figure 4). After multiple comparison correction, posthoc tests revealed that the P3 mean amplitude was significantly greater for the no‐go condition in the Parietal_Z, Parietal_Right, and Central_Z clusters (*p* < 0.001, *p* < 0.01, *p* < 0.001, respectively). There was no significant condition difference in the Parietal_Left cluster, so the difference between the go and no‐go P3 mean amplitude of the three other clusters tested were averaged together and used in further analyses.

### 3.3 Cortical source reconstruction

**FIGURE 5:**
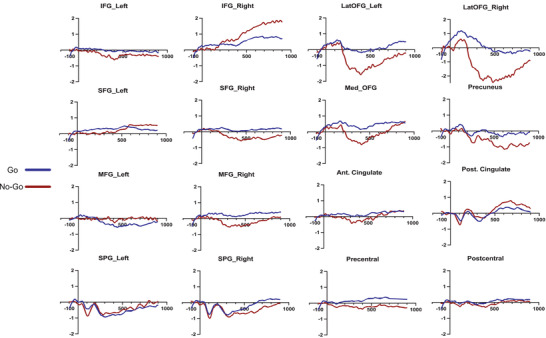
Grand‐averaged SVD values elicited in regions of interest. *Note*. IFG, Inferior frontal gyrus; Lat, Lateral; OFG, Orbitofrontal gyrus; SFG, Superior frontal gyrus; Med, Medial; MFG, Medial frontal gyrus, Ant., Anterior; Post., Posterior; SPG, Superior parietal gyrus

To investigate the neural generators of the ERP components of interest, source analysis was conducted. Average ERP waveforms from each of our 16 ROIs were computed and are displayed in Figure 5. The grand‐averaged ERP waveforms generated from these regions suggest that the superior parietal gyrus and posterior cingulate exhibit canonical N2 and P3 responses, and thus may play a role in generating these components. The lateral and medial orbitofrontal gyri, right inferior frontal gyrus, and precuneus appeared to have different ERP response patterns to the go versus no‐go condition, suggesting these regions may play a role in successful inhibitory control.

**FIGURE 6:**
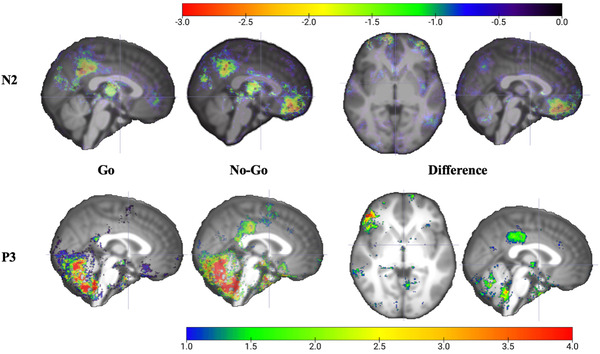
3D Displays of grand‐averaged SVD amplitudes during go/no‐go task. *Note*. The average SVD values for the go trials, no‐go, and the difference between conditions are displayed on an age‐appropriate MRI template. The top row shows activations at the peak latency values for the N2 component. The bottom row shows activations at the peak latency values for the P3 component

Figure 6 shows a 3 D rendering of the average SVD for go and no‐go stimuli plotted on an average template of available participant MRIs at the peak latency values for the N2 and P3 components. Visual inspection suggests increased activation in prefrontal regions for no‐go trials than go trials at the peak latency of the N2. Increased activation in cingulate regions was apparent for no‐go versus go trials at the peak latency of the P3. Overall, the SVD activations seen in this sample appear to be largely consistent with previous studies that have identified frontal, parietal, and cingulate regions as the neural generators of the N2 and P3 components.

### 3.4 Associations among ERP responses, behavioral performance, and IQ

Bivariate Pearson correlations were computed to test whether ERP components were associated with behavioral performance on the go/no‐go task or IQ (Table 1). The N2 peak amplitude was not associated with accuracy, reaction time, or IQ. The N2 peak latency was significantly associated with accuracy on the go/no‐go task (*r*(152) = 0.16, *p* = 0.04), but not reaction time or IQ. The P3 mean amplitude was significantly related to IQ (*r*(152) = 0.28, *p* < 0.001), but not to accuracy or reaction time.

**TABLE 1:** Correlations between ERP components, behavioral go/no‐go performance, and IQ

**Measure**	N2 peak amp.	N2 latency	P3 mean amp.	Accuracy	Reaction time	IQ
N2 peak amp.	**—**					
N2 latency	−0.08	**—**				
P3 mean amp.	0.01	−0.10	**—**			
Accuracy	−0.11	0.16^*^	0.06	**—**		
Reaction time	0.07	0.01	−0.09	−0.13	**—**	
Full‐scale IQ	0.04	−0.05	0.28^***^	0.28^***^	0.23^***^	**—**

*Note*. Pearson correlation coefficients are presented. All ERP measures are normalized differences between the go and no‐go components.

Abbreviations: Amp., Amplitude; IQ, Intelligence quotient.

^*^ *p* < .05;

^**^ *p* < .01;

^***^ *p* < .001.

Correlations between behavioral performance on the go/no‐go task and IQ were also calculated to assess whether behavioral correlates of inhibitory control were related to overall cognitive functioning. IQ was found to be significantly correlated with both accuracy (*r*(152) = 0.28, *p* < 0.001) and reaction time (*r*(152) = ‐0.23, *p* < 0.001).

### 3.5 Associations with risk factors

Multiple linear regressions were performed to examine whether measures of early adversity were associated with ERP components of interest, behavioral performance on the go/no‐go task, or IQ. For each outcome variable, a model with the poverty index, HAZ, maternal stress index, and psychosocial adversity score (CPAS score) was tested to examine multiple co‐occurring risk factors (see Table 2). The regression models for the N2 peak amplitude, N2 latency, P3 mean amplitude, and reaction time all revealed no significant associations with any risk factor, when controlling for all other measures. Psychosocial adversity was significantly related to mean accuracy, after controlling for the other risk factors in the model (*B* = −0.001, *t*(142) = −3.79, *p* < 0.001), such that children who experienced higher levels of psychosocial adversity tended to have lower accuracy on the go/no‐go task. Psychosocial adversity was also significantly negatively associated with IQ after controlling for other risk factors (*B* = −0.117, *t*(142) = −3.09, *p* < 0.01).

**TABLE 2:** Regressions testing associations of risk factors with inhibitory control and IQ

	Outcome variable					
Risk factors	N2 peak amp.	N2 latency	P3 mean amp.	Accuracy	Reaction time	IQ
Intercept	−0.5627*	0.0587	0.8020*	0.8661***	0.7435***	93.4924***
	(−0.2593)	(−0.0401)	(−0.3593)	(−0.0246)	(−0.0254)	(2.5639)
Poverty index	−0.0185	0.0022	−0.0255	0.0015	−0.0001	−0.2261
	(−0.016)	(−0.0025)	(−0.0222)	(−0.0015)	(−0.0016)	(0.1581)
HAZ	−0.0465	0.0062	0.0748	0.0102	−0.0115	1.3954
	(−0.0814)	(−0.0126)	(−0.1129)	(−0.0077)	(−0.008)	(0.8052)
Maternal stress index	0.0082	−0.0007	0.0056	0.0001	−0.0006	0.0552
	(−0.0049)	(−0.0008)	(−0.0068)	(−0.0005)	(−0.0005)	(0.0483)
Psychosocial adversity	0.0015	0.0001	−0.0067	**−0.0014** ***	0.0006	**−0.1174** **
	(−0.0038)	(−0.0006)	(−0.0053)	(−0.0004)	(−0.0004)	(0.0380)
R^2^	0.0314	0.0106	0.0393	0.1199	0.0431	0.1488
Adj. R^2^	0.0041	−0.0172	0.0123	0.0951	0.0161	0.1248
Num. obs.	147	147	147	147	147	147

*Note*. Unstandardized beta coefficients are presented. Standard errors presented in parentheses below.

Abbreviations: Amp., Amplitude; HAZ, Height‐for‐age z‐score; IQ, Intelligence quotient.

^*^ *p* < .05;

^**^ *p* <.01;

^***^ *p* < .001.

## 4 DISCUSSION

In the present study we aimed to explore whether previously identified neurobiological mechanisms underlying inhibition are generalizable to 5‐year‐old children living in Dhaka, Bangladesh. We investigated whether neural correlates of inhibitory control differed by children's exposure to adverse factors (e.g., poverty, malnutrition, maternal health, psychosocial adversity), by testing relations among early adversity, neural mechanisms underlying inhibitory control, and cognitive outcomes.

Overall, children displayed expected patterns of behavioral performance on the go/no‐go task, exhibiting lower accuracy and shorter reaction times for no‐go trials than go‐trials. These findings are in line with prior studies in 5‐year‐old children in HICs (St. John et al., 2019; Abdul Rahman et al., 2017), suggesting that the behavioral inhibition task elicited a comparable cognitive challenge in Dhaka as in high‐income contexts at this age. Furthermore, these findings also align with behavioral performance seen in 8‐ and 10‐ to 11‐year‐old samples in LMICs, and support the feasibility of administering the go/no‐go task to younger children in low‐resource contexts (Loman et al., 2013; McDermott et al., 2012).

In our sample, children showed a more pronounced (more negative) N2 peak amplitude for the go condition than the no‐go condition in three of four electrode clusters tested. This pattern of response contradicts findings from 5‐ to 10‐year‐old children in HICs showing a more pronounced N2 for no‐go trials than go trials (Abdul Jonkman, 2006; Rahman et al., 2017). Notably, our N2 peak results are consistent with a study of adolescents involved in the U.S. child welfare system. Maltreated participants in this study had more pronounced N2 amplitudes to no‐go trials versus go trials, whereas their nonmaltreated peers exhibited the opposite, expected pattern; these findings suggest that the go/no‐go condition difference in the N2 peak amplitude may serve as a marker of the effect of maltreatment (Bruce & Kim, 2020). In the current analyses, we examined the CPAS (psychosocial adversity questionnaire) full scale score, which includes subscales related to neglect, abuse, community violence, etc.; we do not find an association between the CPAS and the N2 (or P3) at 5 years old, and therefore cannot lend support for the N2 as a marker of the impact of psychosocial adversity from this analysis. However, future research should investigate whether specific maltreatment‐related subscales of the CPAS (e.g., harsh discipline and neglect, verbal abuse and family conflict) are associated with the N2 component at 5 years of age in an LMIC.

In contrast to the N2 peak, the N2 latency exhibited patterns seen in the existing literature, with a longer latency to the no‐go trials than the go trials in frontal regions. This condition difference is in concordance with numerous previous studies in both HICs and LMICs throughout childhood (Hoyniak, 2017; Liao et al., 2017), although some studies have found no difference in the N2 latency for conditions requiring versus not requiring inhibition in 8‐ to 11‐year‐old samples (Loman et al., 2013; McDermott et al., 2012). The longer latency exhibited by participants during behavioral inhibition lends support to the N2 latency as a marker of inhibitory control. Similarly, our P3 findings are in line with previous work with 5‐ to 10‐year old showing that children have larger amplitudes for no‐go trials requiring inhibition than go trials requiring no inhibition (St. Jonkman, 2006; St. John et al., 2019). Together, the observed responses indicate that behavioral inhibition and certain neural correlates of inhibitory control appear to operate similarly in this sample as in HICs, but patterns of N2 peak responses may not be universally generalizable and should be investigated further in diverse contexts.

Cortical source reconstruction of the ERP responses suggested that prefrontal, cingulate, and parietal regions may be neural generators of the N2 and P3, in line with our hypotheses. For instance, the precuneus and posterior cingulate appeared to exhibit differences in source waveforms during go versus no‐go trials, which aligns with previous evidence suggesting that these areas play a role in focused attention and stimulus discrimination beginning in infancy (Xie et al., 2019). The consistency between our findings and previous studies suggests that the neural generators of the N2 and P3 may be similar in this context as compared in HICs. This could indicate that inhibition operates similarly in diverse contexts, with possible differences in the N2 peak amplitude warranting further investigation. Further studies should explore how adverse factors may specifically relate to activations in prefrontal, cingulate, and parietal regions.

We did not find the expected associations between ERP responses and behavioral performance on the go/no‐go task. We did find that the N2 latency was positively correlated with accuracy, but this association was only marginally significant. Neither the N2 peak amplitude nor P3 mean amplitude were related to accuracy or reaction time, in contrast to previous studies that have established brain‐behavior relationships in similar paradigms (St. Brydges et al., 2014; St. John et al., 2019). This suggests that while the N2 and P3 ERP components differ for trials requiring inhibition versus no inhibition, these brain differences do not serve as reliable, specific predictors of inhibitory control in this context at 5 years of age. Many brain regions in the fronto‐parietal and ventral‐attention networks are recruited during behavioral inhibition. High‐level attentional and decision‐making processes also contribute to children's ability to control their impulses (Diamond, 2013; Zhang et al., 2017). Therefore, future studies should explore a wider range of neural measures in addition to the N2 and P3 to more comprehensively examine the complex processes involved in behavioral inhibition.

The P3 was found to be positively correlated with children's IQ scores, suggesting that although the P3 did not specifically index behavioral inhibition, it could serve as a useful predictor of overall cognitive functioning. In addition to the P3, children's accuracy and reaction time during the go/no‐go task were also associated with IQ scores. The interrelatedness between behavioral correlates of inhibition and overall cognitive skills has also been demonstrated in 2‐ to 4‐year‐old children in Pakistan (Obradović et al., 2019). These findings suggest that behavioral and ERP data from simple EF tasks such as the go/no‐go task, could provide useful biomarkers of specific aspects of EF development, and complement more comprehensive, resource‐intensive cognitive assessments such as the WPPSI. Future work should explore the directionality, predictive validity, and developmental timing of the relationship between inhibition and general cognition.

We hypothesized that children's exposure to a range of risk factors would be related to both correlates of inhibitory control and general cognitive functioning. Unexpectedly, we did not find any associations between aspects of adversity and our ERP components of interest. In contrast, studies in both HICs and LMICs have found SES, exposure to neglect, and maltreatment to be associated with neural correlates of inhibitory control throughout childhood (Bruce & Kim, 2020; Loman et al., 2013; McDermott et al., 2012; St. John et al., 2019). Given these prior studies and our findings of atypical N2 peak responses, it is possible that the adverse factors examined do in fact impact the N2 and/or P3 at the group‐level but there are insufficient individual differences in our sample to detect this relationship due to participants’ high rates of extreme poverty. Future research will examine ERP correlates from a higher‐income sample also living in Dhaka, Bangladesh to explore whether associations between adversity and inhibition are revealed in a sample with a wider variation of risk exposure. Additionally, it is important to note that measures of poverty, malnutrition, and maternal mental health were collected at 36 months of age, while the go/no‐go ERP paradigm was administered at 60 months of age. Thus, while we do not find any longitudinal associations between these measures of adversity and ERP components of interest, there may be concurrent associations at 60 months that we were unable to explore given the available data in the current study.

Although we did not find that risk factors examined in this study (poverty, malnutrition, maternal stress, and psychosocial adversity) specifically relate to the N2 and P3, we did find that children who experienced higher levels of psychosocial adversity had lower accuracy and IQ scores, on average. Thus, psychosocial adversity is related to both inhibition and overall cognition, but differences in the N2 and P3 do not appear to underlie this association. The CPAS measure of psychosocial adversity was collected concurrently with the ERP measures at 60 months, in contrast to the other measures of adversity collected at 36 months. Therefore, the CPAS measure may serve as a stronger signal of current exposure level instead of assuming consistent longitudinal exposure. Future studies should investigate additional potential neural mechanisms that could explain how psychosocial adversity becomes biologically embedded. The excellent time resolution of ERPs allows for investigation of indices of cognitive processes at the scale of milliseconds. However, individual ERP components such as the N2 and P3 represent very specific correlates of cognition. Inhibitory control relies on complex frontal‐striatal circuitry, and thus there are many other candidate neural correlates of inhibition reflecting broader processing that could be driving the association between psychosocial adversity and cognition. For instance, future research using EEG may investigate theta band power or frontal and frontal‐parietal connectivity, which have been implicated in cognitive processes related to EF (Buzzell et al., 2017). Additionally, fMRI research can offer higher spatial resolution to verify the brain regions implicated in inhibitory control in the current study.

We found that psychosocial adversity is associated with cognitive outcomes, whereas SES, malnutrition, and maternal mental health are not. Many interventions in LMICs focus on improving SES, nutrition, or maternal mental health, and these factors are widely considered as important to supporting healthy early childhood development (Fernald & Hidrobo, 2011; Husain et al., 2021; Ocansey et al., 2019; Prado et al., 2020). We may not find associations with certain adversity measures for several reasons. As mentioned above, there was a 2‐year lag between our collection of SES, malnutrition, and maternal mental health measures and the cognitive assessment; therefore, possible fluctuations in exposure level during this lag that may explain the limited associations found. Additionally, there may be insufficient variability or multicollinearity among certain adversity variables that made it difficult to detect associations between specific variables and WPPSI outcomes. While there may be relations between these adversity variables and cognition that we were unable to detect in this study, our finding that psychosocial adversity is concurrently associated with cognitive outcomes provides motivation for the proliferation of intervention strategies that seek to mitigate child maltreatment, intimate partner violence, domestic abuse, and other adverse psychosocial experiences.

This study demonstrates the utility of employing neuroimaging and behavioral methods in LMICs to explore the development, mechanisms, and environmental influences of executive functions. However, the current study does have several limitations. First, we measure only a fraction of the multitude of early experiences that could potentially affect inhibitory control and cognitive skills. We do not measure possible protective factors that may promote resiliency such as parental responsivity or cognitive stimulation. We also were limited to exploring only stimulus‐locked ERP components due to insufficient number of incorrect responses, and thus were not able to investigate the role of the error‐related negativity in inhibitory control. Additionally, this current study relied on the WPPSI as a measure of overall cognitive development, which was originally designed for high‐income, Western contexts. The WPPSI was rigorously adapted to the Bangladeshi context, but this measure may still not be well‐suited to assessing cognitive outcomes in this sample, introducing bias and noise. In follow‐up studies, we will explore how inhibitory control is related to school readiness and reading abilities to investigate the role of executive functioning in children's academic outcomes.

Overall, the current study indicates that some, but not all, correlates of inhibitory control in this unique sample of 5‐year‐old children are consistent with findings from studies in high‐income settings. Although the N2 and P3 responses were not found to be directly associated with the adverse factors examined in this study, the influence of early adversity on the neural mechanisms underlying inhibitory control should not be ruled out; instead, future studies should investigate additional brain measures that are sensitive to environmental inputs and recruit a sample with wider variability in exposure to risk factors. The associations seen between correlates of inhibition and IQ indicate that the go/no‐go paradigm may be a valuable tool in predicting children's cognitive outcomes. Our study demonstrates the utility of deploying neuroimaging in LMICs to elucidate relevant and valid markers of inhibition in young children in diverse contexts. Deepening our understanding of the biological underpinnings of cognitive development in LMICs will aid in the earlier identification of children at risk of delays or deficits, leading to the design and implementation of more effective intervention strategies to promote children's success and well‐being.

## CONFLICT OF INTEREST

The authors declare no conflicts of interest.

## Supporting information

Supporting Information

## Data Availability

The data used in this study are available, upon reasonable request, from Charles A. Nelson (Charles_Nelson@harvard.edu).
