# Supplementary material for: Neural correlates of inhibitory control and associations with cognitive outcomes in Bangladeshi children exposed to early adversities
Source: Dev Sci. 2022 Feb 22;25(5):e13245. doi: 10.1111/desc.13245 (PMC9393202; doi:10.1111/desc.13245)
Supplement: Supplementary file 1 — Supporting Information [file DESC-25-e13245-s001.docx]

**Appendix**

**Supplemental Table 1** Channel clusters and corresponding HGSN electrodes used in analyses

| **Cluster** | **Electrodes** |
| --- | --- |
| Frontal_Left | 19, 23, 26, 27, 28, 33, 34 |
| Frontal_Z | 4, 5, 10, 11, 12, 16, 18, 19 |
| Frontal_Right | 2, 3, 116, 117, 122, 123, 124 |
| Central_Z | 7, 31, 55, 80, 106 |
| Parietal_Left | 47, 51, 52, 53, 59, 60 |
| Parietal_Z | 61, 62, 67, 77, 78 |
| Parietal_Right | 85, 86, 91, 92, 97, 98 |

**Supplemental Table 2** *Descriptive statistics of risk factors and cognitive outcomes*

| **Variable** | **Mean** | ***SD*** | **Min.** | **Max.** |
| --- | --- | --- | --- | --- |
| Poverty index | 12.83 | 5.56 | 2 | 24 |
| Income (USD/day/person) | 2.1  8 | 2.11 | 0.20 | 10.67 |
| Maternal education (years) | 5.80 | 3.82 | 3 | 10 |
| HAZ | -1.33 | 1.04 | -3.48 | 2.16 |
| Maternal stress index | 52.28 | 17.23 | 25 | 121 |
| Psychosocial adversity | 39.33 | 21.66 | 0 | 123 |
| Full-scale IQ | 86.94 | 9.33 | 67 | 115 |
| Performance IQ | 86.64 | 9.46 | 65 | 114 |
| Verbal IQ | 87.44 | 7.86 | 72 | 114 |

*Note.* Abbreviations: USD, United States Dollars; HAZ, Height-for-age Z-score; IQ, Intellectual Quotient; SD, Standard deviation; Min., Minimum; Max., Maximum
